# Supplementary figures and images for: Risk of severe influenza infection in women with a history of pregnancy complications: A longitudinal cohort study
Source: PLoS One. 2024 Nov 13;19(11):e0313653. doi: 10.1371/journal.pone.0313653 (PMC11560043; doi:10.1371/journal.pone.0313653)

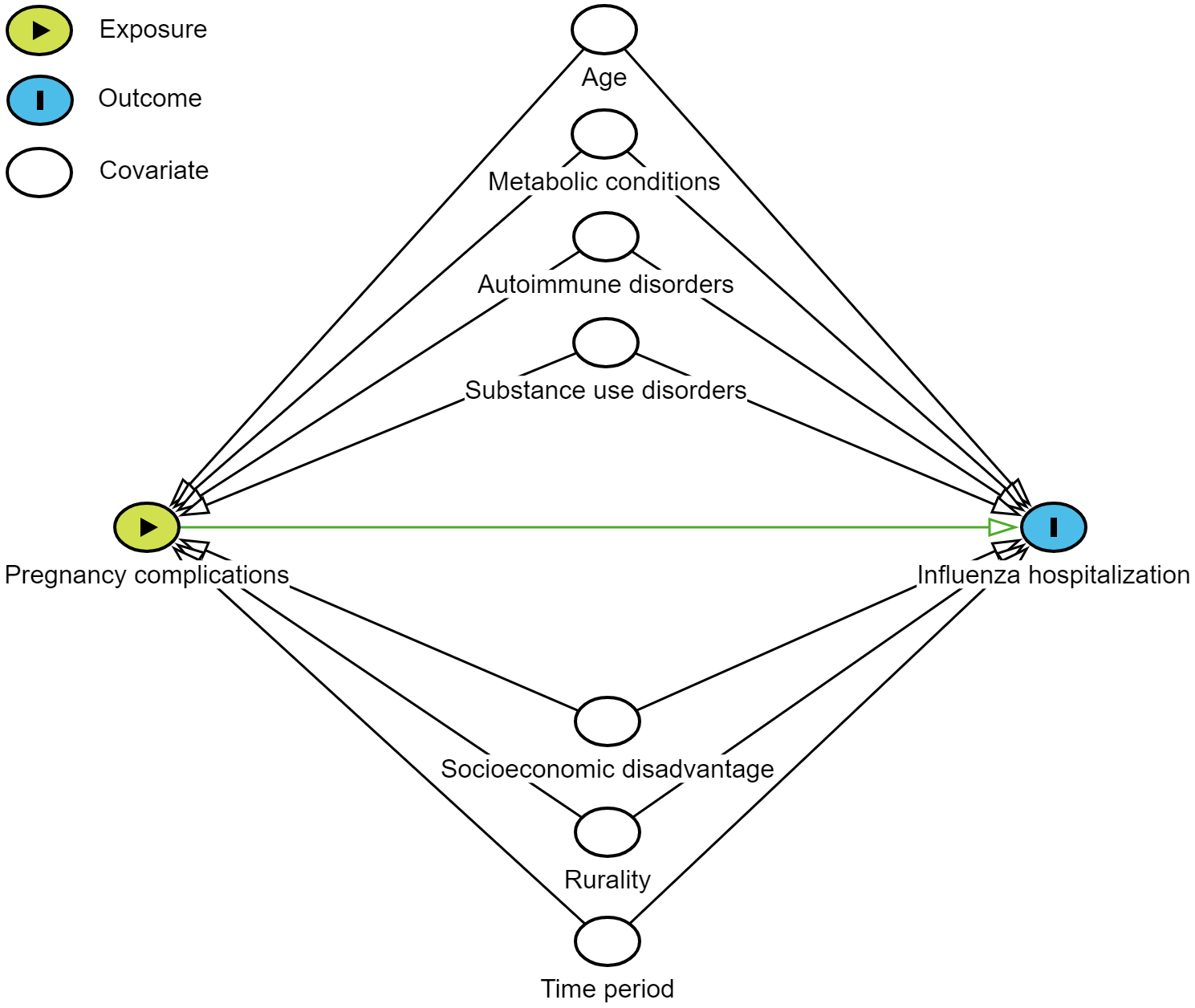

Supplement: S1 Fig — The DAG was prepared using DAGitty v3.1. (TIF) [file pone.0313653.s001.tif]
