# Supplementary material for: Risk of severe influenza infection in women with a history of pregnancy complications: A longitudinal cohort study
Source: PLoS One. 2024 Nov 13;19(11):e0313653. doi: 10.1371/journal.pone.0313653 (PMC11560043; doi:10.1371/journal.pone.0313653)
Supplement: S1 Table — (DOCX) [file pone.0313653.s002.docx]

**S1 Table. Diagnostic codes for influenza and related complications.^a^**

|  | International Classification of Diseases-9 / Canadian Classification of Diagnostic, Therapeutic, and Surgical Procedures (before 2006) | International Classification of Diseases-10 / Canadian Classification of Health Interventions (beginning 2006) |
| --- | --- | --- |
| Influenza | 487, 488 | J09-J11 |
| Intubation | 10.04, 10.05, 43.19, 43.21 | 1.GZ.31.CA-ND, 1.GZ.31.CR-ND |
| Adult respiratory distress syndrome | 518.52, 518.82 | J80 |
| Superimposed pneumonia | 480-486 | J12-J18 |

^a^Intensive care unit admission was identified by field codes in the data registry.
