# Supplementary material for: Risk of severe influenza infection in women with a history of pregnancy complications: A longitudinal cohort study
Source: PLoS One. 2024 Nov 13;19(11):e0313653. doi: 10.1371/journal.pone.0313653 (PMC11560043; doi:10.1371/journal.pone.0313653)
Supplement: Attachment — Submitted filename: PONE-D-23-39142R1_Amer_Response to reviews.docx [file pone.0313653.s003.docx]

**RESPONSE TO REVIEWS**

**Editor Comments:**

**E.1. Please ensure that your manuscript meets PLOS ONE's style requirements, including those for file naming. The PLOS ONE style templates can be found at**

[**https://journals.plos.org/plosone/s/file?id=wjVg/PLOSOne_formatting_sample_main_body.pdf**](https://journals.plos.org/plosone/s/file?id=wjVg/PLOSOne_formatting_sample_main_body.pdf) **and** 
[**https://journals.plos.org/plosone/s/file?id=ba62/PLOSOne_formatting_sample_title_authors_affiliations.pdf**](https://journals.plos.org/plosone/s/file?id=ba62/PLOSOne_formatting_sample_title_authors_affiliations.pdf)

Response: We ensured that we followed style requirements.

**E.2. Note from Emily Chenette, Editor in Chief of PLOS ONE, and Iain Hrynaszkiewicz, Director of Open Research Solutions at PLOS:**

**Did you know that depositing data in a repository is associated with up to a 25% citation advantage (**[**https://doi.org/10.1371/journal.pone.0230416**](https://doi.org/10.1371/journal.pone.0230416)**)? If you’ve not already done so, consider depositing your raw data in a repository to ensure your work is read, appreciated and cited by the largest possible audience. You’ll also earn an Accessible Data icon on your published paper if you deposit your data in any participating repository (**[**https://plos.org/open-science/open-data/#accessible-data**](https://plos.org/open-science/open-data/#accessible-data)**).**

Response: We confirm that the raw data are available from the Institut de la statistique du Québec repository and can be accessed upon request following standard procedures (https://www.stat.gouv.qc.ca/recherche/#/accueil).

**E.3. Thank you for stating the following financial disclosure:**

**"This work was supported by the Canadian Institutes of Health Research (grant number PJT-162300) and Fonds de recherche du Québec-Santé (grant number 296785)."**

**Please state what role the funders took in the study. If the funders had no role, please state: "****The funders had no role in study design, data collection and analysis, decision to publish, or preparation of the manuscript." 
If this statement is not correct you must amend it as needed.**

**Please include this amended Role of Funder statement in your cover letter; we will change the online submission form on your behalf.**

Response: We added the role of the funders in the cover letter: “*the funders had no role in study design, data collection and analysis, decision to publish, or preparation of the manuscript*”.

**E.4. Please note that funding information should not appear in the Acknowledgments section or other areas of your manuscript. We will only publish funding information present in the Funding Statement section of the online submission form. Please remove any funding-related text from the manuscript.**

Response: We deleted the funding information from the manuscript.

**E.5. We note that you have indicated that there are restrictions to data sharing for this study. For studies involving human research participant data or other sensitive data, we encourage authors to share de-identified or anonymized data. However, when data cannot be publicly shared for ethical reasons, we allow authors to make their data sets available upon request. For information on unacceptable data access restrictions, please see** [**http://journals.plos.org/plosone/s/data-availability#loc-unacceptable-data-access-restrictions**](http://journals.plos.org/plosone/s/data-availability#loc-unacceptable-data-access-restrictions)**.**

**Before we proceed with your manuscript, please address the following prompts:**

**a) If there are ethical or legal restrictions on sharing a de-identified data set, please explain them in detail (e.g., data contain potentially identifying or sensitive patient information, data are owned by a third-party organization, etc.) and who has imposed them (e.g., a Research Ethics Committee or Institutional Review Board, etc.). Please also provide contact information for a data access committee, ethics committee, or other institutional body to which data requests may be sent.**

**b) If there are no restrictions, please upload the minimal anonymized data set necessary to replicate your study findings to a stable, public repository and provide us with the relevant URLs, DOIs, or accession numbers. Please see** [**http://www.bmj.com/content/340/bmj.c181.long**](http://www.bmj.com/content/340/bmj.c181.long) **for guidelines on how to de-identify and prepare clinical data for publication. For a list of recommended repositories, please see** [**https://journals.plos.org/plosone/s/recommended-repositories**](https://journals.plos.org/plosone/s/recommended-repositories)**. You also have the option of uploading the data as Supporting Information files, but we would recommend depositing data directly to a data repository if possible.**

**Please update your Data Availability statement in the submission form accordingly.**

Response: We confirm that due to legal restrictions we cannot share the data. The data are owned by a third party, the Quebec Health Ministry. Any researcher wishing to access the data must make a formal request following standard procedures. We clarified the data availability statement as follows: “*The underlying data used in the study are owned by the Quebec Health Ministry and are available upon request from the Institut de la statistique repository following standard access procedures, https://www.stat.gouv.qc.ca/recherche/#/accueil*”.

**E.6. Please review your reference list to ensure that it is complete and correct. If you have cited papers that have been retracted, please include the rationale for doing so in the manuscript text, or remove these references and replace them with relevant current references. Any changes to the reference list should be mentioned in the rebuttal letter that accompanies your revised manuscript. If you need to cite a retracted article, indicate the article’s retracted status in the References list and also include a citation and full reference for the retraction notice.**

Response: We confirm that the reference list is complete and correct.

**E.7. Thank you for submitting your manuscript to PLOS ONE!
After careful consideration, we feel that the goal of this study is relevant and it has technical rigor, but does not fully meet PLOS ONE’s publication criteria as it currently stands. Therefore, we invite you to submit a revised version of the manuscript that addresses the points raised during the review process – specially in the Methods’ section.**

Response: We thank the Editors and Reviewers and have carefully incorporated their recommendations. We hope you will be pleased with the revised manuscript.

**Reviewer #1:**

**R1.1. An interesting work with a significant number, but that includes different social responses to the disease throughout the time period. It would be important to clarify that over such a long period of time the approach strategies vary equally and are not the same.**

Response: We thank the Reviewer. We confirm that patients admitted towards the end of the study may be more likely to be vaccinated. However, we have no reason to suspect that influenza vaccination levels differ between patients with and without pregnancy complications. We added the limitation that “*We could not account for changes in influenza vaccination over time, although any changes are likely to have affected patients with and without a history of pregnancy complications equally*” (page 17, lines 2-4).

**Reviewer #2:**

**It is my pleasure to review this study. This study examined the association of obstetric complications with influenza hospitalization for up to 32 years among more than 1 million pregnant women. In general, this is a well-conducted and well-written paper. I have a few comments that the authors may consider.**

**R2.1. Covariate selection: the authors may consider using DAGs**

Response: We thank the Reviewer and added a DAG in the supplement. We added that “*We used a directed acyclic graph to identify factors that could potentially influence the risk of pregnancy complications and influenza morbidity (S1 Fig)*” (page 6, lines 5-6).

**R2.2. There is no mention of how to calculate cumulative incidence. The K-M method without considering competing events may lead to a biased estimate of cumulative incidence.**

Response: We used the cumulative incidence function to account for competing events (Lin 2012). We clarified that “*We computed the incidence per 1,000 women after 32 years of follow-up* *using the cumulative incidence function, accounting for deaths as a competing event [16]*” (page 6, lines 17-19).

Lin G, et al. Analyzing survival data with competing risks using SAS® software. Orlando, FL: SAS Institute Inc.; 2012.

**R2.3. How about assumptions of proportional hazard? What approach has been used to evaluate this assumptions?**

Response: We added that “*We evaluated the proportional hazards assumption using log (−log survival) curves*” (page 7, lines 6-7).

**R2.4. As one may experience multiple complications during one pregnancy, the authors may consider the dose-relationship between the number of pregnancy complications and risks of influenza.**

Response: We agree with the Reviewer that patients could have more than one complication, including more than one pregnancy with multiple complications. As it was challenging to address multiple pregnancies with multiple complications in a time-to-event analysis, we added the recommendation that “*Further research is needed to determine the extent to which the number of pregnancy complications affects the risk of influenza hospitalization later in life*” (page 17, lines 8-9).
